# Overall, anti-malarial, and non-malarial effect of intermittent preventive treatment during pregnancy with sulfadoxine-pyrimethamine on birthweight: a mediation analysis

**DOI:** 10.1016/S2214-109X(20)30119-4

**Published:** 2020-06-17

**Authors:** Michelle E Roh, Feiko O ter Kuile, Francois Rerolle, M Maria Glymour, Stephen Shiboski, Roly Gosling, Julie Gutman, Abel Kakuru, Meghna Desai, Richard Kajubi, Anne L'Ianziva, Moses R Kamya, Grant Dorsey, R Matthew Chico

**Affiliations:** a Department of Epidemiology and Biostatistics, University of California, San Francisco, CA, USA; b Malaria Elimination Initiative, Global Health Group, University of California, San Francisco, CA, USA; c Division of HIV, Infectious Diseases, and Global Medicine, Department of Medicine, University of California, San Francisco, CA, USA; d Liverpool School of Tropical Medicine, Liverpool, UK; e Malaria Branch, Division of Parasitic Diseases and Malaria, Center for Global Health, US Centers for Disease Control and Prevention, Atlanta, GA, USA; f Infectious Diseases Research Collaboration, Kampala, Uganda; g Centers for Disease Control and Prevention, Kisumu, Kenya; h School of Medicine, Makerere University College of Health Sciences, Kampala, Uganda; i Department of Disease Control, Faculty of Infectious and Tropical Disease, London School of Hygiene & Tropical Medicine, London, UK

## Abstract

**Background:**

Trials of intermittent preventive treatment (IPTp) of malaria in pregnant women that compared dihydroartemisinin-piperaquine with the standard of care, sulfadoxine-pyrimethamine, showed dihydroartemisinin-piperaquine was superior at preventing malaria infection, but not at improving birthweight. We aimed to assess whether sulfadoxine-pyrimethamine shows greater non-malarial benefits for birth outcomes than does dihydroartemisinin-piperaquine, and whether dihydroartemisinin-piperaquine shows greater antimalarial benefits for birth outcomes than does sulfadoxine-pyrimethamine.

**Methods:**

We defined treatment as random assignment to sulfadoxine-pyrimethamine or dihydroartemisinin-piperaquine before pooling individual participant-level data from 1617 HIV-uninfected pregnant women in Kenya (one trial; n=806) and Uganda (two trials; n=811). We quantified the relative effect of treatment on birthweight (primary outcome) attributed to preventing placental malaria infection (mediator). We estimated antimalarial (indirect) and non-malarial (direct) effects of IPTp on birth outcomes using causal mediation analyses, accounting for confounders. We used two-stage individual participant data meta-analyses to calculate pooled-effect sizes.

**Findings:**

Overall, birthweight was higher among neonates of women randomly assigned to sulfadoxine-pyrimethamine compared with women assigned to dihydroartemisinin-piperaquine (mean difference 69 g, 95% CI 26 to 112), despite placental malaria infection being lower in the dihydroartemisinin-piperaquine group (relative risk [RR] 0·64, 95% CI 0·39 to 1·04). Mediation analyses showed sulfadoxine-pyrimethamine conferred a greater non-malarial effect than did dihydroartemisinin-piperaquine (mean difference 87 g, 95% CI 43 to 131), whereas dihydroartemisinin-piperaquine conferred a slightly larger antimalarial effect than did sulfadoxine-pyrimethamine (8 g, −9 to 26), although more frequent dosing increased the antimalarial effect (31 g, 3 to 60).

**Interpretation:**

IPTp with sulfadoxine-pyrimethamine appears to have potent non-malarial effects on birthweight. Further research is needed to evaluate monthly dihydroartemisinin-piperaquine with sulfadoxine-pyrimethamine (or another compound with non-malarial effects) to achieve greater protection against malarial and non-malarial causes of low birthweight.

**Funding:**

Eunice Kennedy Shriver National Institute of Child Health and Human Development, Bill & Melinda Gates Foundation, and Worldwide Antimalarial Resistance Network.

## Introduction

In sub-Saharan Africa, malaria infection during pregnancy is a major cause of low birthweight. For pregnant women, red blood cells infected with *Plasmodium falciparum* sequester in the placenta, causing inflammatory cellular responses that lead to increased risk of preterm delivery (<37 gestational weeks) and intrauterine growth restriction, which are both causes of low birthweight (<2500 g).^1^, ^2^, ^3^ To prevent adverse consequences of malaria infection, WHO recommends the provision of intermittent preventive treatment (IPTp) with sulfadoxine-pyrimethamine to all pregnant women living in areas of moderate-to-high malaria transmission, administered at scheduled antenatal visits from the second trimester to delivery.^4^

Parasite resistance to sulfadoxine-pyrimethamine in eastern and southern Africa has led researchers to evaluate alternative drug regimens for IPTp. Dihydroartemisinin-piperaquine remains the most promising candidate, given its long-acting prophylactic effect and highly efficacious antimalarial activity. To date, three trials^5^, ^6^, ^7^ in areas of high sulfadoxine-pyrimethamine resistance have shown that dihydroartemisinin-piperaquine is well-tolerated and more effective in preventing malaria infection than is sulfadoxine-piperaquine. However, this effect did not translate into better birth outcomes.^5^, ^6^, ^7^ A plausible explanation for these results is that these studies lacked sufficient statistical power to detect differences in birth outcomes, as most of them were powered to detect differences in malaria outcomes, which were more prevalent. An alternative explanation is that sulfadoxine-pyrimethamine, via the broad-spectrum antimicrobial activity of sulfadoxine, improves birth outcomes through mechanisms that are independent of its antimalarial activity (ie, via non-malarial mechanisms), and in these studies, the non-malarial effect has offset the greater antimalarial effect of dihydroartemisinin-piperaquine on birth outcomes. Studies support this alternative hypothesis, suggesting IPTp with sulfadoxine-pyrimethamine remains protective against low birthweight risk in areas with low malaria transmission^8^ or high parasite resistance to sulfadoxine-pyrimethamine.^9^, ^10^, ^11^

**Research in context**

**Evidence before the study**

We searched ClinicalTrials.gov and PubMed for original articles using the search terms “intermittent preventive treatment” AND “sulfadoxine-pyrimethamine” AND “malaria in pregnancy” OR “non-malarial effect” OR “bacterial vaginosis” OR “reproductive tract infections” OR “sexually transmitted infections”. No language or time restrictions were used in this search. We identified two observational studies, done in malarious regions of Zambia and Burkina Faso, and one editorial that addressed the potential non-malarial effect of sulfadoxine-pyrimethamine. Both observational studies found intermittent preventive treatment with sulfadoxine-pyrimethamine in pregnant women (IPTp) had a dose-dependent effect on birth outcomes, with one study reporting that IPTp with sulfadoxine-pyrimethamine was associated with reduced odds of sexually transmitted and reproductive tract infections, and overall adverse birth outcomes. The editorial, which cited a study done in a low malaria prevalence (<1%) setting of Zambia, reported that IPTp with sulfadoxine-pyrimethamine conferred a protective effect on birth outcomes. However, none of these studies differentiated between the non-malarial and antimalarial effects of IPTp with sulfadoxine-pyrimethamine on adverse birth outcomes.

**Added value of this study**

To our knowledge, this is the first study to use mediation analysis on individual-participant data from randomised controlled trials of IPTp to deconstruct the non-malarial and antimalarial effects of sulfadoxine-pyrimethamine relative to dihydroartemisinin-piperaquine. Dihydroartemisinin-piperaquine is one of the most promising candidates to replace sulfadoxine-pyrimethamine in IPTp regimens. Our findings show that IPTp with sulfadoxine-pyrimethamine confers a greater non-malarial effect on birthweight than does IPTp with dihydroartemisinin-piperaquine, but IPTp with dihydroartemisinin-piperaquine has moderately greater antimalarial effects on birthweight than does IPTp with sulfadoxine-pyrimethamine if given at monthly intervals.

**Implications of all the available evidence**

In areas with high parasite resistance to sulfadoxine-pyrimethamine, IPTp with sulfadoxine-pyrimethamine could be more beneficial in improving birthweight through preventing the non-malarial causes of adverse pregnancy outcomes compared with IPTp with dihydroartemisinin-piperaquine. However, IPTp with dihydroartemisinin-piperaquine, when given monthly, has moderately greater antimalarial effects on birthweight than does IPTp with sulfadoxine-pyrimethamine. The combination of dihydroartemisinin-piperaquine plus a partner compound that confers protection against the non-malarial causes of lower birthweight (eg, sulfadoxine-pyrimethamine) could increase the public health effect of IPTp more than dihydroartemisinin-piperaquine or sulfadoxine-pyrimethamine alone. However, further IPTp trials are needed to validate whether the combination of dihydroartemisinin-piperaquine and sulfadoxine-pyrimethamine (or any combination that targets both the malarial and non-malarial causes of lower birthweight) would be safe and efficacious.

To test this alternative hypothesis, we aimed to use data from three randomised trials in Kenya and Uganda to assess whether sulfadoxine-pyrimethamine shows greater non-malarial benefits for birth outcomes than does dihydroartemisinin-piperaquine, and whether dihydroartemisinin-piperaquine shows greater antimalarial benefits for birth outcomes than does sulfadoxine-pyrimethamine. We used mediation analyses^12^, ^13^ to estimate the non-malarial and antimalarial effects of these two treatments. Mediation analysis is an epidemiological method that uses statistical modelling to examine quantitatively the extent to which certain intermediate variables mediate the overall effect of a treatment on an outcome. Mediation analysis is done by prespecifying a mediator and estimating the effect that a treatment has on an outcome either indirectly (via the mediator) or directly (via the non-mediated pathway). In this study, the term indirect effect is defined as the effect of IPTp on birthweight that is attributed to preventing placental malaria (ie, antimalarial effect). The term direct effect is defined as the effect of IPTp on birthweight that is not attributed to preventing placental malaria (ie, non-malarial effect). Here, we present analyses of the relative overall, indirect, and direct effects of these two IPTp regimens on birth outcomes.

## Methods

### Study population

We collected individual participant-level data from three trials in Siaya County, Kenya (Kenya-STOPMiP),^5^ Tororo District, Uganda (Uganda-BC1),^6^ and Busia District, Uganda (Uganda-BC3).^7^ In Siaya County, around 96% of parasites carry the quintuple antifolate mutation (*pfdhfr* 51I, 59R, and 108N and *pfdhps* 437G and 540E) and 5·8% have the sextuple mutation (*pfdhps A581G*).^9^ In Tororo, Uganda, around 78% of parasites carry the quintuple mutation, whereas none have the sextuple mutation.^14^ No data were available on *pfdhf/pfdhps* mutations in Busia, although Tororo and Busia are adjacent districts.

Trial eligibility was restricted to HIV-uninfected pregnant women who were resident in the study region or health facility catchment area with no history of receiving IPTp during their current pregnancy.

In the Kenya-STOPMiP trial,^5^ women between 16 and 32 gestational weeks of pregnancy were enrolled and randomly assigned to receive IPTp with dihydroartemisinin-piperaquine, IPTp with sulfadoxine-pyrimethamine, or intermittent screening and treatment (ISTp) with dihydroartemisinin-piperaquine. Women assigned to IPTp groups received IPTp at enrolment and then at each subsequent antenatal visit at intervals of 4–6 weeks.

In the Ugandan studies,^6^, ^7^ women between 12 and 20 gestational weeks of pregnancy were enrolled. In Uganda-BC1,^6^ women were randomly assigned to receive either IPTp with sulfadoxine-pyrimethamine every 8 weeks, IPTp with dihydroartemisinin-piperaquine every 8 weeks, or IPTp with dihydroartemisinin-piperaquine every 4 weeks. In Uganda-BC3,^7^ women were randomised to either IPTp with sulfadoxine-pyrimethamine or IPTp with dihydroartemisinin-piperaquine every 4 weeks. Women assigned to IPTp every 8 weeks began IPTp at 20 gestational weeks of pregnancy, whereas women assigned to IPTp every 4 weeks began IPTp at 16 or 20 gestational weeks of pregnancy, depending on their gestational age at enrolment.

For all studies, each dose of sulfadoxine-pyrimethamine was three tablets of 500 mg sulfadoxine and 25 mg of pyrimethamine given as a single dose. In Kenya-STOPMiP,^5^ dosing of dihydroartemisinin-piperaquine was based on bodyweight at enrolment (two, three, or four tablets of 40 mg dihydroartemisinin and 320 mg piperaquine per day for bodyweights of 24·0–35·9 kg, 36·0–74·9 kg; or ≥75·0 kg, respectively) and given once a day for 3 days. In the Ugandan studies,^6^, ^7^ each dose of dihydroartemisinin-piperaquine was three tablets of 40 mg dihydroartemisinin and 320 mg piperaquine given once a day for 3 days. Single-dose sulfadoxine-pyrimethamine and the first dose of dihydroartemisinin-piperaquine were administered under direct observation at the clinic, and the second and third doses of dihydroartemisinin-piperaquine were self-administered at home. The Ugandan trials were placebo-controlled such that all participants received a three-dose course. Participants in Kenya were visited at home 2 days after enrolment to verify drug adherence, and every fifth participant was visited at home on subsequent visits. For the Ugandan studies, standardised assessments were done to determine adherence.

Our mediation analysis included women who had singleton livebirths, received at least one IPTp dose, and a known status of either past or active placental malaria infection. Placental malarial infection was determined by including women who either had a histopathological assessment of placental malaria using placenta tissue or women who tested positive for placental malaria by either microscopy, loop-mediated isothermal amplification, or PCR methods using placental blood. Women were excluded if they were assigned to the Kenya-STOPMiP ISTp group, assigned to the Uganda-BC1 IPTp with dihydroartemisinin-piperaquine every 4 weeks group, or had an unknown status of either past or active placental malaria (ie, missing placental histopathology results and negative for placental malaria by microscopy and molecular methods).

Ethics approvals were granted by the Kenya Medical Research Institute, Makerere University School of Biomedical Sciences, the Uganda National Council for Science and Technology, the US Centers for Disease Control and Prevention, and the University of California, San Francisco.

### Measurement of treatment, mediator, and confounders

We defined treatment as random assignment to IPTp with either sulfadoxine-pyrimethamine or dihydroartemisinin-piperaquine. The mediator in our analysis was defined as the presence of previous or active placental malaria infection. A woman was determined to have a previous or active placental malaria infection if she had pigment or parasites in her placenta determined by histopathology of the placental tissue^15^ or if she tested positive for parasites by microscopy or molecular methods in her placental blood. Peripherally-detected malaria infection was considered a potential mediator (appendix 2 p 1) but we found that, in our sample of women, parasitaemia without the presence of placental malaria was not associated with lower birthweight, whereas women with placental malaria, regardless of whether they had peripherally-detected malaria, were more likely to have a baby with a lower birthweight.

Confounding variables were identified a priori based on causal assumptions represented in a directed acyclic graph (appendix 2 p 2). Because of treatment randomisation, confounders were limited to those that affected mediator–outcome associations. These included gestational age at enrolment, maternal age, maternal parasitaemia at enrolment, gravidity, education, and household wealth. Gravidity was dichotomised as primigravidae (first pregnancy) or multigravidae (one or more previous pregnancies). Household wealth was reported as tertiles and calculated using principal components analysis of common household items.

### Outcomes

The primary outcome was a continuous measure of birthweight at delivery measured in g. Secondary outcomes were low birthweight (<2500 g) and preterm delivery (<37 gestational weeks). Further details on measurement of these outcomes are reported in the trials.^5^, ^6^, ^7^

### Statistical analysis

We used causal mediation analysis^16^, ^17^, ^18^ to deconstruct the crude differences in birth outcomes between IPTp regimens (ie, overall treatment effect) into the difference in birth outcomes between IPTp regimens that is mediated by preventing placental malaria (ie, indirect or antimalarial effect) and the difference in birth outcomes between IPTp regimens that is not mediated by preventing placental malaria infection (ie, direct or non-malarial effect; appendix 2 pp 2–3).

We estimated crude differences in birth outcomes between IPTp regimens using linear or log-binomial regression models with random assignment as the sole predictor. For mediation analyses, we used the mediation R package^19^ to estimate indirect and direct effects (appendix 2 p 3). We ran separate models to specify the dependence of placental malaria and birth outcomes based on treatment and prespecified confounders (as described in the assumed causal graph; appendix 2 p 2). Predicted values from these models were used in a Monte-Carlo framework to calculate indirect and direct effect estimates and corresponding 95% CIs, which we report as mean differences for birthweight and relative risks for low birthweight and preterm delivery.

For all models, treatment–gravidity and treatment–mediator interaction terms were tested wherever possible and incorporated if the p values (p_interaction_) of these terms were less than 0·10. We modelled continuous predictors as three-knot restricted cubic splines if the p value of the *F* test for the joint-effect of the non-linear components was less than 0·05. CIs around mediation effect estimates were generated for each study with a quasi-Bayesian approach using 1000 simulations. Effect modification by gravidity of indirect and direct effect estimates was tested using the test.modmed() function^19^ with corresponding p values reported as p_difference_. For the mediator and primary outcome (placental malaria and birthweight, respectively), we report effect estimates separately for each study and by gravidity, regardless of whether there was evidence of a statistical interaction. Analyses of secondary outcomes (low birthweight and preterm delivery) were not reported separately by gravidity as they were relatively uncommon.

We generated pooled-effect estimates using two-stage individual participant data meta-analyses. Individual participant data were used to derive effect estimates for each study and combined using a DerSimonian-Laird random-effects model from the meta R package.^20^ Between-study heterogeneity was measured using the *I*^2^ statistic. Analyses were done using Stata version 14.0 and R version 3.5.0.

### Role of the funding source

The funders of the study had no role in study design, data collection, data analysis, data interpretation, or writing of the report. The corresponding author had full access to all the data in the study and had final responsibility for the decision to submit for publication.

## Results

**Figure 1:**
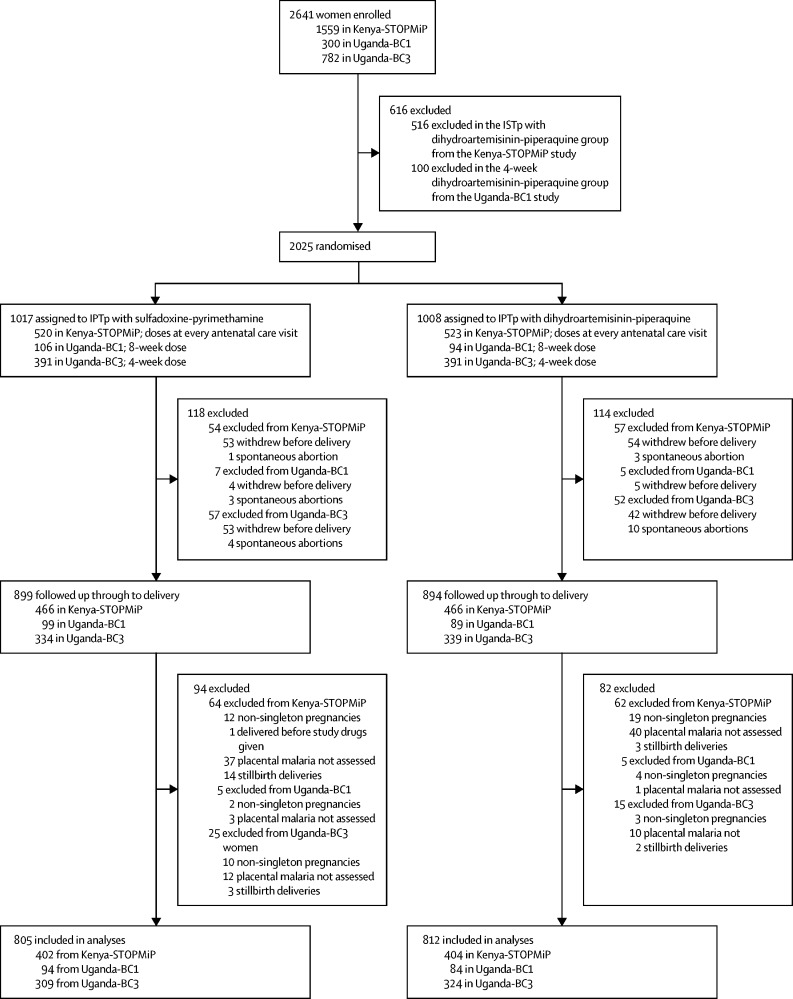
Flowchart of participants from the Kenya-STOPMiP, Uganda-BC1, and Uganda-BC3 IPTp trials who were included in our primary analysis IPTp=intermittent preventive treatment.

Our primary analysis included 1617 women—806 from Kenya-STOPMiP,^5^ 178 from Uganda-BC1,^6^ and 633 from Uganda-BC3^7^ (figure 1). 1024 (39%) of 2641 women enrolled across the three studies were excluded for the following reasons: 516 women randomly assigned to non-IPTp group; 100 women enrolled in the Uganda-BC1 monthly dihydroartemisinin-piperaquine group and did not have a monthly sulfadoxine-pyrimethamine group as a study-specific comparator; 211 women withdrew from the study before delivery; 21 women had a spontaneous abortion, 22 women had a stillbirth, and 50 women had a non-singleton pregnancy; 103 women did not have placental malaria assessed; and one woman did not receive any study drug. The proportions of women excluded from each category were similar between IPTp groups (p>0·05).

Baseline characteristics and the number of IPTp doses given were similar between IPTp groups (table). In Kenya-STOPMiP,^5^ all women participating in random home visits adhered to taking their second and third IPTp doses. Self-reported adherence to second and third IPTp doses was 99% in Uganda-BC1^6^ and 98% in Uganda-BC3.^7^

**Figure 2:**
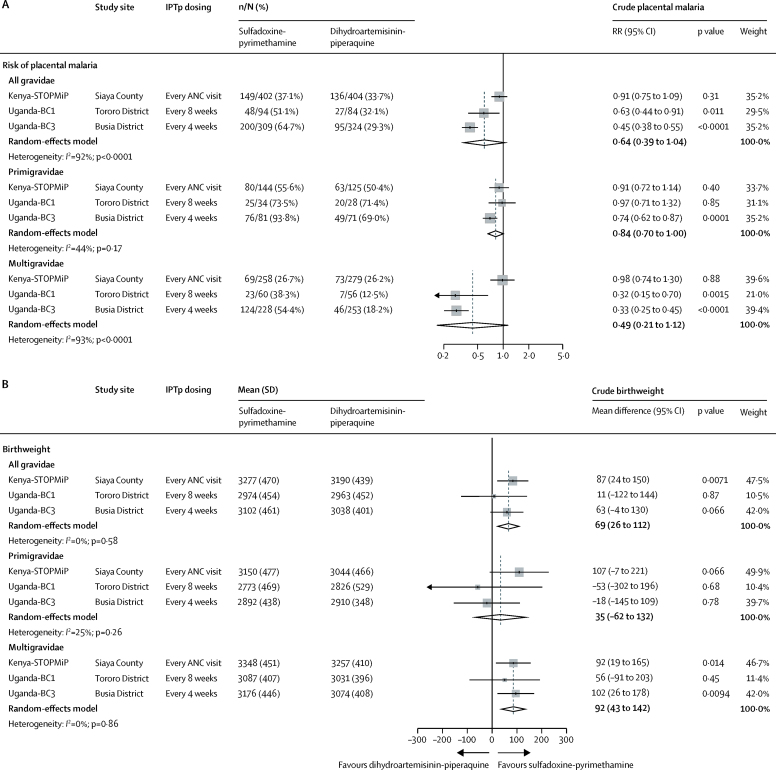
Crude differences in placental malaria risk (A) and birthweight (B) between IPTp groups by study and gravidity subgroup ANC=antenatal care. IPTp=intermittent preventive treatment. RR=relative risk.

In the pooled analysis, dihydroartemisinin-piperaquine was associated with a lower risk of placental malaria infection compared with sulfadoxine-pyrimethamine, but this finding did not reach statistical significance (relative risk [RR] 0·64, 95% CI 0·39–1·04). There was substantial heterogeneity between studies (*I*^2^=92%; p<0·0001) and effects differed between primigravidae and multigravidae in the Ugandan studies (figure 2). In Kenya-STOPMiP, dihydroartemisinin-piperaquine was not associated with a substantially lower risk of placental malaria (RR 0·91, 95% CI 0·75–1·09) and effects were similar between primigravidae and multigravidae (p_interaction_=0·68). In the Ugandan studies, dihydroartemisinin-piperaquine was associated with a significantly lower risk of placental malaria compared with sulfadoxine-pyrimethamine (RR 0·63, 95% CI 0·44–0·91 in Uganda-BC1 and 0·45, 0·38–0·55 in Uganda-BC3) and effects were larger in multigravidae compared with primigravidae (figure 2; p_interaction_=0·0095 for Uganda-BC1 and p_interaction_=<0·0001).

**Figure 3:**
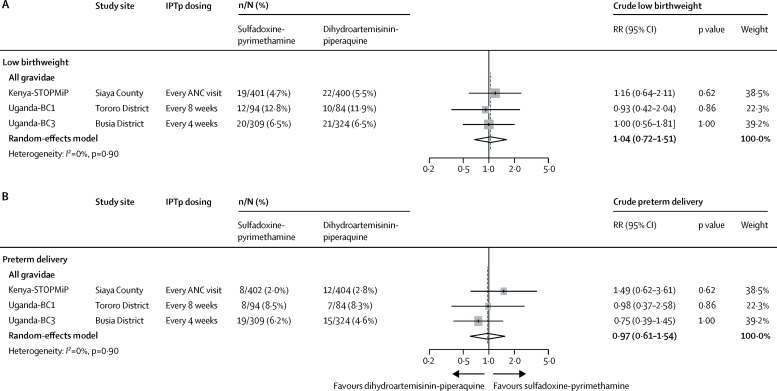
Crude differences in low birthweight (A) and preterm delivery (B) between IPTp groups by study IPTp=intermittent preventive treatment. ANC=antenatal care. RR=relative risk.

Although dihydroartemisinin-piperaquine was associated with a lower risk of placental malaria compared with sulfadoxine-pyrimethamine, neonates born to mothers randomly assigned to dihydroartemisinin-piperaquine had lower birthweight (mean difference in pooled analysis 69 g, 95% CI 26–112; figure 2). Effect estimates were similar between studies (*I*^2^=0%; p=0·58). In Kenya-STOPMiP, the mean difference was 87 g (95% CI 24–150) and effects were similar between primigravidae and multigravidae (p_interaction_=0·82). In Ugandan studies, sulfadoxine-pyrimethamine was not associated with a significantly higher birthweight compared with dihydroartemisinin-piperaquine and there was insufficient evidence that effects differed by gravidity (p_interaction_ Uganda-BC1=0·43; p_interaction_ Uganda-BC3=0·13). However, risks of low birthweight (ie, <2500 g) and preterm delivery did not significantly differ between IPTp regimens, overall or for any of the three individual studies (figure 3).

**Figure 4:**
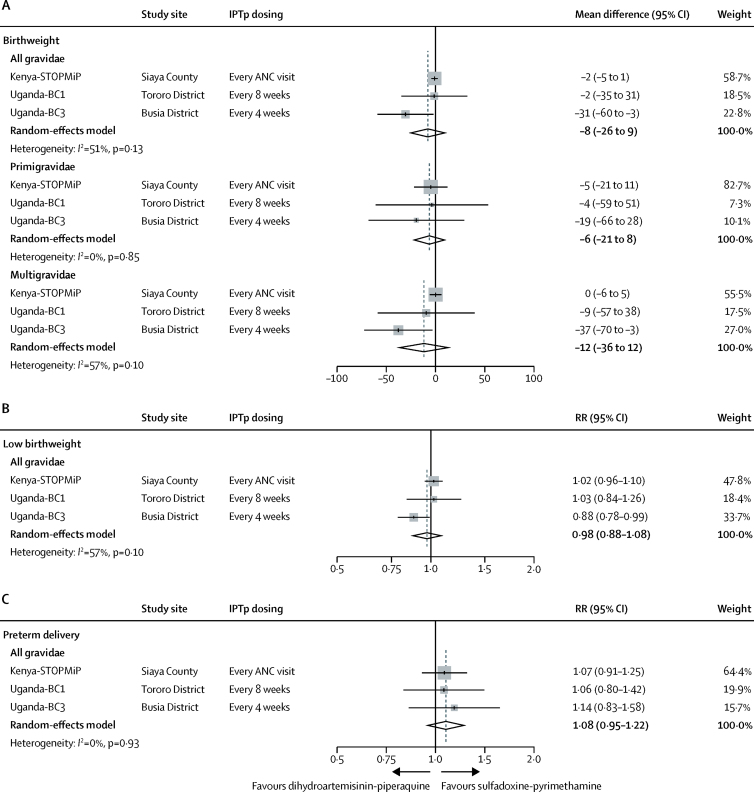
Effect of IPTp regimens on birthweight (A), low birthweight (B), and preterm delivery (C), mediated by placental malaria Birthweight mediation effect estimates are presented by gravidity subgroup. ANC=antenatal care. IPTp=intermittent preventive treatment. RR=relative risk.

In mediation analyses, dihydroartemisinin-piperaquine did not show a significantly larger antimalarial effect on birthweight than did sulfadoxine-pyrimethamine in the pooled analysis (mean difference 8 g, 95% CI −9 to 26), although effect estimates varied between studies (*I*^2^=51%; p=0·13; figure 4). The effect size was larger and statistically significant in the Uganda-BC3 study, in which IPTp was given monthly (mean difference 31 g, 95% CI 3 to 60). In the other studies, in which most women received three or less IPTp doses (table 1), the mean difference was 2 g, with CIs that included the null. We found no evidence that antimalarial effects differed between primigravidae and multigravidae in any of the three studies (p_difference_>0·63).

**Table:** Characteristics of study populations by study and randomised treatment group

		**Kenya-STOPMiP**^5^		**Uganda-BC1**^6^		**Uganda-BC3**^7^	
		Sulfadoxine-pyrimethamine (n=402)	Dihydroartemisinin-piperaquine (n=404)	Sulfadoxine-pyrimethamine (n=94)	Dihydroartemisinin-piperaquine (n=84)	Sulfadoxine-pyrimethamine (n=309)	Dihydroartemisinin-piperaquine (n=324)
**At enrolment**							
Age at enrolment (years)		23·7 (5·9)	23·3 (5·4)	21·5 (3·7)	22·2 (4·4)	24·0 (6·0)	24·0 (5·7)
Weight (kg)		61·6 (9·0)	61·7 (9·3)	55·5 (6·9)	55·6 (6·9)	56·0 (7·8)	55·8 (7·8)
Gestational age (weeks)		22·7 (19·7–26·3)	22·9 (19·9–26·1)	14·9 (13·4–16·9)	14·9 (14·0–16·6)	15·7 (13·4–17·9)	15·1 (13·4–17·1)
Wealth index tertiles							
	Lowest	133 (33%)	124 (31%)	36 (38%)	28 (33%)	103 (33%)	112 (35%)
	Middle	139 (35%)	133 (33%)	27 (29%)	32 (38%)	102 (33%)	110 (34%)
	Highest	128 (32%)	145 (36%)	31 (33%)	24 (29%)	104 (34%)	102 (31%)
Level of education							
	None or primary	241 (60%)	233 (58%)	75 (80%)	67 (80%)	238 (77%)	244 (75%)
	Secondary and beyond	159 (40%)	167 (42%)	19 (20%)	17 (20%)	71 (23%)	80 (25%)
Gravidity							
	Primigravidae (first pregnancy)	144 (36%)	125 (31%)	34 (36%)	28 (33%)	81 (26%)	71 (22%)
	Multigravidae (second or later pregnancy)	258 (64%)	279 (69%)	60 (64%)	56 (67%)	228 (74%)	253 (78%)
Slept under a net during previous night		288 (72%)	292 (72%)	81 (86%)	77 (92%)	104 (34%)	108 (33%)
Maternal parasitaemia*		126 (33%)	120 (31%)	52 (55%)	50 (60%)	254 (82%)	257 (79%)
Maternal haemoglobin (g/dL)		10·6 (1·5)	10·6 (1·5)	11·8 (1·5)	11·9 (1·1)	11·4 (1·4)	11·4 (1·2)
**Following enrolment**							
Number of intermittent preventive treatment doses received							
	1	20 (5%)	11 (3%)	0	0	0	0
	2	158 (39%)	179 (44%)	6 (6%)	5 (6%)	1 (1%)	0
	3	110 (27%)	114 (28%)	88 (94%)	79 (94%)	2 (1%)	1 (1%)
	4	85 (21%)	68 (17%)	0	0	6 (2%)	7 (2%)
	5	25 (6%)	29 (7%)	0	0	80 (26%)	79 (24%)
	6	4 (1%)	3 (1%)	0	0	155 (50%)	152 (47%)
	7	0	0	0	0	65 (22%)	85 (26%)

Data are mean (SD), median (IQR), or n (%). Values many not sum to totals because of missing data.

* Malaria parasitaemia assessed by loop-mediated isothermal amplification in the Uganda-BC1 study and by quantitative polymerase chain reaction in the Kenya-STOPMiP and Uganda-BC3 studies.

Antimalarial effects on low birthweight showed similar patterns to birthweight. In pooled analyses, the antimalarial effects on low birthweight were similar between IPTp groups (RR 0·98, 95% CI 0·88–1·08), although effect estimates were heterogeneous between studies (*I*^2^=57%; p=0·10; figure 4). Compared with the Kenya-STOPMiP and Uganda-BC1 studies, which showed null differences between IPTp regimens (RR 1·02, 95% CI 0·96–1·10 and 1·03, 0·84–1·26, respectively), the Uganda-BC3 study (in which IPTp was dosed monthly) showed that dihydroartemisinin-piperaquine conferred a greater and statistically significant antimalarial effect on low birthweight than did sulfadoxine-pyrimethamine (0·88, 0·78–0·99). Antimalarial effects on preterm delivery risk were similar between IPTp regimens (1·08, 95% CI 0·95–1·22) in the pooled analysis and across the three studies (*I*^2^=0%; p=0·93; figure 4).

**Figure 5:**
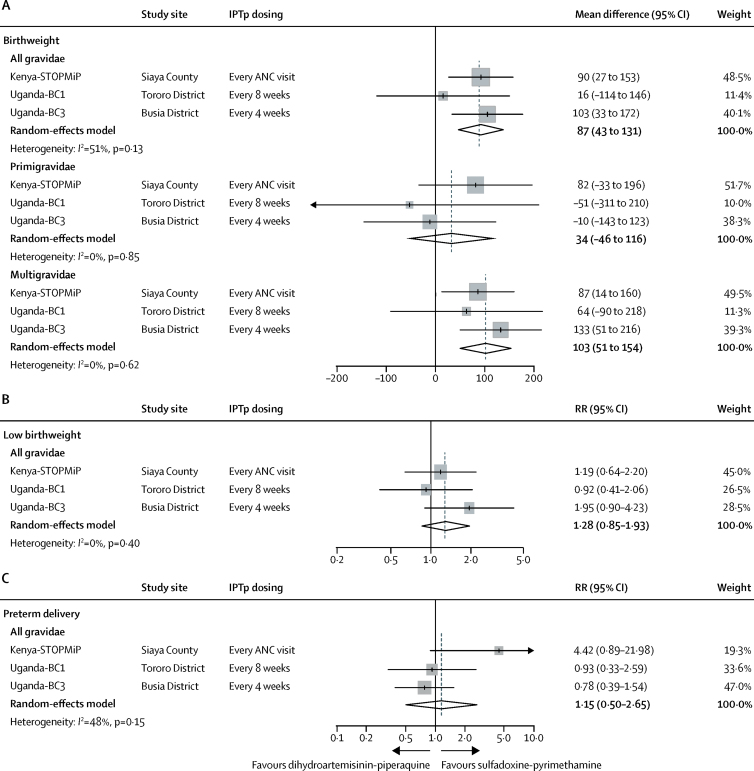
Effect of IPTp regimens on birthweight (A), low birthweight (B), and preterm delivery (C), not mediated by placental malaria Birthweight mediation effect estimates are presented by gravidity subgroup. ANC=antenatal care. IPTp=intermittent preventive treatment. RR=relative risk.

In the pooled analysis, sulfadoxine-pyrimethamine conferred a greater non-malarial effect on birthweight than did dihydroartemisinin-piperaquine (mean difference 87 g, 95% CI 43 to 131; figure 5). Effects were similar across studies (*I*^2^=0%; p=0·51) and we found no evidence that the non-malarial effects differed between primigravidae and multigravidae in the Kenya-STOPMiP and Uganda-BC1 study (p_difference_>0·33). In the Uganda-BC3 study, sulfadoxine-pyrimethamine conferred a greater non-malarial effect than did dihydroartemisinin-piperaquine in multigravidae, but not in primigravidae (mean difference 133 g, 95% CI 51 to 216 *vs* −10 g, −143 to 123, respectively; p_difference_=0·094).

The non-malarial effect on low birthweight had a similar relationship to the continuous measure of birthweight (figure 5). In the pooled analysis, sulfadoxine-pyrimethamine conferred a 22% (or 100×[1–1/1·28]) greater non-malarial effect on low birthweight risk compared with dihydroartemisinin-piperaquine (RR 1·28, 95% CI 0·85–1·93), although CIs included the null. Low birthweight effect estimates were similar between studies (*I*^2^=0%; p=0·40).

In the pooled analysis, sulfadoxine-pyrimethamine was associated with a 13% (or 100×[1–1/1·15]) greater non-malarial effect on preterm delivery risk than was dihydroartemisinin-piperaquine (RR 1·15, 95% CI 0·50–2·65), although CIs around all effect estimates included the null (figure 5). Effects varied between studies (*I*^2^=48%; p=0·15), particularly between Kenyan and Ugandan studies.

We did sensitivity analyses to test the robustness of our effect estimates. First, we restricted the definition of our mediator to active placental infections only, as past infections include those that might have been present before study enrolment. Dihydroartemisinin-piperaquine was associated with a lower risk of active infections compared with sulfadoxine-pyrimethamine (appendix 2 p 4), and we found that mediation effect estimates on birthweight did not substantially differ from the primary analyses (appendix 2 p 5). Second, we tested the robustness of our effect estimates to unmeasured mediator-outcome confounding (appendix 2 p 6). We found that the strength of an unmeasured confounder would have to be implausibly large to explain away our observed non-malarial effect, but not our antimalarial effect estimate.

## Discussion

By pooling data from three randomised controlled trials, we found evidence that IPTp influences birthweight through both antimalarial and non-malarial mechanisms. Crude analyses of data from these trials showed that, despite the substantially larger protective effect of dihydroartemisinin-piperaquine on placental malaria, birthweight was higher for neonates of women randomly assigned to sulfadoxine-pyrimethamine. We did mediation analyses to investigate this seemingly paradoxical relationship. We found, via mechanisms mediated by malaria prevention, that monthly IPTp with dihydroartemisinin-piperaquine (as observed in the Uganda-BC3 study) was associated with a modest, but significant increase in birthweight (31 g) compared with sulfadoxine-pyrimethamine. By contrast, there was little difference in the antimalarial effect on birthweight between IPTp groups in studies with less frequent IPTp dosing (ie, Kenya-STOPMiP and Uganda-BC1). We found that, via mechanisms not mediated by malaria, sulfadoxine-pyrimethamine was associated with a significant increase in birthweight (87 g) compared with dihydroartemisinin-piperaquine, and this effect was similar across studies. Antimalarial effects on preterm delivery risk did not follow birthweight or low birthweight trends, suggesting the mechanism by which IPTp affects birthweight might be via promotion of intrauterine fetal growth, rather than timing of delivery. We observed some evidence in Uganda that non-malarial effects were greater in multigravidae, which might reflect a greater attributable fraction of non-malarial causes of low birthweight compared with primigravidae, for whom malaria might be a more predominant cause of low birthweight.

Although we do not know the exact non-malarial mechanisms by which sulfadoxine-pyrimethamine is improving birthweight, it is likely that the antibiotic properties of sulfadoxine are, at least partly, responsible for these observed effects.^8^, ^21^, ^22^ Sulfadoxine belongs to a group of agents (sulfonamides) that have been previously used to treat *Trichomonas vaginalis,*^23^ *Gardnerella vaginalis* (a bacterium associated with bacterial vaginosis),^24^ *Neisseria gonorrhoeae,*^25^ and *Chlamydia trachomatis.*^26^ These infections are prevalent among pregnant women in east Africa (range 3·7–50·8%).^27^ Although sulfadoxine-pyrimethamine is unlikely to cure these infections, antenatal dosing has been shown to reduce adverse pregnancy outcomes among women who had these non-malarial infections at antenatal booking.^28^ There are probably other mechanisms at play, which warrant future study, including those affected by the broad-spectrum antibacterial activity of sulfadoxine.^29^ For example, sulfadoxine might alter the maternal intestinal or vaginal microbiome to stimulate fetal growth^21^, ^22^ or modulate maternal immunity, similar to effects described for the related antifolate combination trimethoprim-sulfamethoxazole.^11^ Although identifying the specific mechanisms underlying the non-malarial effect of sulfadoxine-pyrimethamine were not within the scope of this study, our findings show that IPTp with sulfadoxine-pyrimethamine could be used to prevent the non-malarial causes of lower birthweight, which might be just as important as, if not more so, than preventing placental malaria infection.

In the Uganda-BC3 study, 457 (73%) of 633 women received at least six IPTp doses, whereas in the Kenya-STOPMiP and Uganda-BC1 study, 592 (73%) of 806 and 178 (100%) of 178 women received three or less IPTp doses, respectively. More frequent dosing could explain why the antimalarial effect of dihydroartemisinin-piperaquine was larger in the Uganda-BC3 study than the other studies. Our findings support the results of the original Uganda-BC1 study,^6^ which found that dihydroartemisinin-piperaquine administered every 4 weeks was associated with lower malaria and adverse birth outcome risk than when administered every 8 weeks. Thus, to take advantage of the full antimalarial benefits of IPTp with dihydroartemisinin-piperaquine, particularly in areas of high sulfadoxine-pyrimethamine resistance or high malaria burden, doses should be given at monthly intervals and as early in the second trimester as possible.

This study has limitations. First, mediation effect estimates might have been subject to unmeasured confounding, although our sensitivity analyses suggest non-malarial effects were fairly robust. Second, placental malaria (mediator) could have been measured with error, which would have probably, on average, have biased the antimalarial and non-malarial effect toward the null. However, a sensitivity analysis using a more specific definition of the mediator (active placental malaria infections) showed similar results. Third, our meta-analysis effect estimates were derived from only three studies and should be interpreted with caution. Finally, our study might have had low statistical power to detect true differences between gravidity subgroups and further studies are needed to support our findings.

In conclusion, mediation analyses enabled us to quantify the greater benefits of sulfadoxine-pyrimethamine against the non-malarial causes of lower birthweight compared with dihydroartemisinin-piperaquine, and the greater benefits of monthly dihydroartemisinin-piperaquine against placental malaria as a cause of lower birthweight compared with sulfadoxine-pyrimethamine. These findings have two important policy implications. First, this study suggests that IPTp with sulfadoxine-pyrimethamine might be beneficial in areas of low malaria transmission, where IPTp is not currently recommended, as long as the prevalence of these non-malarial causes are high. Second, the study suggests that in areas of high sulfadoxine-pyrimethamine resistance or high malaria burden, rather than replacing sulfadoxine-pyrimethamine with dihydroartemisinin-piperaquine, a combination of these two regimens for monthly IPTp administration might be more efficacious in improving birthweight. Future IPTp trials need to validate the efficacy and safety of this combination. Provided IPTp with sulfadoxine-pyrimethamine and dihydroartemisinin-piperaquine is safe and efficacious, this regimen or other combinations that target both malarial and non-malarial causes of lower birthweight (eg, dihydroartemisinin-piperaquine plus azithromycin^30^ or dihydroartemisinin-piperaquine plus metronidazole^31^) might have a greater public health impact than giving either therapy alone.

## Supplementary Materials

French translation of the abstract

## References

[1] Guyatt HL, Snow RW (2004). Impact of malaria during pregnancy on low birth weight in sub-Saharan Africa. Clin Microbiol Rev.

[2] Guyatt HL, Snow RW (2001). The epidemiology and burden of *Plasmodium falciparum*-related anemia among pregnant women in sub-Saharan Africa. Am J Trop Med Hyg.

[3] Sharma L, Shukla G (2017). Placental malaria: a new insight into the pathophysiology. Front Med (Lausanne).

[4] WHO (2013). WHO policy brief for the implementation of intermittent preventive treatment of malaria in pregnancy using sulfadoxine-pyrimethamine (IPTp-SP).

[5] Desai M, Gutman J, L'lanziva A (2015). Intermittent screening and treatment or intermittent preventive treatment with dihydroartemisinin-piperaquine versus intermittent preventive treatment with sulfadoxine-pyrimethamine for the control of malaria during pregnancy in western Kenya: an open-label, three-group, randomised controlled superiority trial. Lancet.

[6] Kakuru A, Jagannathan P, Muhindo MK (2016). Dihydroartemisinin-piperaquine for the prevention of malaria in pregnancy. N Engl J Med.

[7] Kajubi R, Ochieng T, Kakuru A (2019). Monthly sulfadoxine-pyrimethamine versus dihydroartemisinin-piperaquine for intermittent preventive treatment of malaria in pregnancy: a double-blind, randomised, controlled, superiority trial. Lancet.

[8] Stoner MC, Vwalika B, Smid M (2017). Dosage of sulfadoxine-pyrimethamine and risk of low birth weight in a cohort of Zambian pregnant women in a low malaria prevalence region. Am J Trop Med Hyg.

[9] Desai M, Gutman J, Taylor SM (2016). Impact of sulfadoxine-pyrimethamine resistance on effectiveness of intermittent preventive therapy for malaria in pregnancy at clearing infections and preventing low birth weight. Clin Infect Dis.

[10] Chico RM, Cano J, Ariti C (2015). Influence of malaria transmission intensity and the 581G mutation on the efficacy of intermittent preventive treatment in pregnancy: systematic review and meta-analysis. Trop Med Int Health.

[11] van Eijk AM, Larsen DA, Kayentao K (2019). Effect of *Plasmodium falciparum* sulfadoxine-pyrimethamine resistance on the effectiveness of intermittent preventive therapy for malaria in pregnancy in Africa: a systematic review and meta-analysis. Lancet Infect Dis.

[12] VanderWeele TJ (2016). Mediation analysis: a practitioner's guide. Annu Rev Public Health.

[13] Imai K, Keele L, Yamamoto T (2010). Identification, inference and sensitivity analysis for causal mediation effects. Stat Sci.

[14] Conrad MD, Mota D, Foster M (2017). Impact of intermittent preventive treatment during pregnancy on *Plasmodium falciparum* drug resistance-mediating polymorphisms in Uganda. J Infect Dis.

[15] Rogerson SJ, Hviid L, Duffy PE, Leke RF, Taylor DW (2007). Malaria in pregnancy: pathogenesis and immunity. Lancet Infect Dis.

[16] Pearl J (2001). Direct and indirect effects. Proceedings of the seventeenth conference on uncertainty in artificial intelligence; 2001.

[17] VanderWeele T (2015). Explanation in causal inference: methods for mediation and interaction.

[18] Robins JM, Greenland S (1992). Identifiability and exchangeability for direct and indirect effects. Epidemiology.

[19] Tingley D, Yamamoto T, Hirose K, Keele L, Imai K (2014). Mediation: R package for causal mediation analysis. https://cran.r-project.org/web/packages/mediation/vignettes/mediation.pdf.

[20] Kontopantelis E, Reeves D (2010). metaan: random-effects meta-analysis. Stata J.

[21] Gutman J, Slutsker L (2017). Intermittent preventive treatment with sulfadoxine-pyrimethamine: more than just an antimalarial?. Am J Trop Med Hyg.

[22] Dingens AS, Fairfortune TS, Reed S, Mitchell C (2016). Bacterial vaginosis and adverse outcomes among full-term infants: a cohort study. BMC Pregnancy Childbirth.

[23] Angelucci HM (1945). The treatment of trichomonas vaginitis with a sulfonamide compound. Am J Obstet Gynecol.

[24] Bhattacharyya MN, Jones BM (1980). Haemophilus vaginalis infection. Diagnosis and treatment. J Reprod Med.

[25] Kampmeier RH (1983). Introduction of sulfonamide therapy for gonorrhea. Sex Transm Dis.

[26] Bowie WR, Manzon LM, Borrie-Hume CJ, Fawcett A, Jones HD (1982). Efficacy of treatment regimens for lower urogenital chlamydia trachomatis infection in women. Am J Obstet Gynecol.

[27] Chico RM, Mayaud P, Ariti C, Mabey D, Ronsmans C, Chandramohan D (2012). Prevalence of malaria and sexually transmitted and reproductive tract infections in pregnancy in sub-Saharan Africa: a systematic review. JAMA.

[28] Chico RM, Chaponda EB, Ariti C, Chandramohan D (2017). Sulfadoxine-pyrimethamine exhibits dose-response protection against adverse birth outcomes related to malaria and sexually transmitted and reproductive tract infections. Clin Infect Dis.

[29] Capan M, Mombo-Ngoma G, Makristathis A, Ramharter M (2010). Anti-bacterial activity of intermittent preventive treatment of malaria in pregnancy: comparative in vitro study of sulphadoxine-pyrimethamine, mefloquine, and azithromycin. Malar J.

[30] National Institutes of Health US National Library of Medicine (July 5, 2017). Improving PRegnancy Outcomes with intermittent preVEntive treatment in Africa (IMPROVE). https://clinicaltrials.gov/ct2/show/NCT03208179.

[31] National Institute of Health US National Library of Medicine (Jan 27, 2020). The ASPIRE trial—Aiming for Safe Pregnancies by reducing malaria and Infections of the ReproductivE tract. https://clinicaltrials.gov/ct2/show/NCT04189744.

