# Supplementary material for: Overall, anti-malarial, and non-malarial effect of intermittent preventive treatment during pregnancy with sulfadoxine-pyrimethamine on birthweight: a mediation analysis
Source: Lancet Glob Health. 2020 Jun 17;8(7):e942–53. doi: 10.1016/S2214-109X(20)30119-4 (PMC7303957; doi:10.1016/S2214-109X(20)30119-4)
Supplement: French translation of the abstract [file mmc1.pdf]

THE LANCET
Global Health

Supplementary appendix 1

This translation in French was submitted by the authors and we reproduce it as supplied. It has not been peer reviewed. *The Lancet*'s editorial processes have only been applied to the original in English, which should serve as reference for this manuscript.

Cette traduction en français a été proposée par les auteurs et nous l'avons reproduite telle quelle. Elle n'a pas été examinée par des pairs. Les processus éditoriaux de *Lancet* n'ont été appliqués qu'à l'original en anglais, ce qui devrait servir de référence à ce manuscrit.

Supplement to: Roh ME, ter Kuile FO, Rerolle F, et al. Overall, anti-malarial, and non-malarial effect of intermittent preventive treatment during pregnancy with sulfadoxine-pyrimethamine on birthweight: a mediation analysis. *Lancet Glob Health* 2020; **8:** e942–53.

Resumé

Introduction

Des essais cliniques de traitement préventif intermittent contre le paludisme (TPIp) chez les femmes enceintes ont comparé la dihydroartémisinine-pipéraquine à la sulfadoxine-pyriméthamine, ce dernier traitement étant aujourd'hui préconisé pour le TPIp. Ces essais ont démontré que la dihydroartémisinine-pipéraquine était supérieur par rapport à la sulfadoxine-pyriméthamine pour la prevention de paludisme, mais non pour l'amélioration du poids des nouveau-nés. Nous avons visé à évaluer si la sulfadoxine-pyriméthamine présentait des avantages non paludéens plus importants pour l'issue des grossesse que la dihydroartémisinine-pipéraquine, et si la dihydroartémisinine-pipéraquine présentait de plus grands avantages que la sulfadoxine-pyriméthamine. antipaludiques pour l'issue des grossesse.

Méthodes

Nous avons défini le traitement par une répartition aléatoire à la sulfadoxine-pyriméthamine ou à la dihydroartémisinine-pipéraquine avant de regrouper les données individuelles des participants de 1,617 femmes enceintes non infectées par le VIH au Kenya (un essai; n = 806) et dans l'Ouganda (deux essais; n = 811). Nous avons quantifié l'effet relatif du traitement sur le poids à la naissance (résultat principal) attribué à la prévention de l'infection paludéenne placentaire (médiateur). Nous avons estimé l'effet antipaludéen (indirect) et l'effet non paludéen (direct) du TPIp sur l'issue des grossesses à l'aide d'analyses de médiation causale, tenant compte des facteurs de confusion. Des meta-analyses en deux étapes ont été effectués pour calculer une estimation d'effet regroupée des essais.

Résultats

Dans l'ensemble, le poids à la naissance était plus élevé chez les nouveau-nés de femmes réparties de manière aléatoire à la sulfadoxine-pyriméthamine par rapport aux femmes réparties à la dihydroartémisinine-pipéraquine (différence moyenne de 69 g, IC à 95% de 26 à 112), malgré l'infection placentaire par le paludisme plus faible dans le groupe dihydroartémisinine-pipéraquine (risque relatif [RR] 0,64, IC 95% 0,39 à 1,04). Les analyses de médiation ont montré que la sulfadoxine-pyriméthamine conférait des effets non paludéens plus importants que la dihydroartémisinine-pipéraquine (différence moyenne 87 g, IC 95% 43 à 131), tandis que la dihydroartémisinine-pipéraquine conférait des effets antipaludiques légèrement plus importants que la sulfadoxine-pyriméthamine (8 g, –9 à 26), bien que des doses plus fréquentes aient augmenté les effets antipaludiques (31 g, 3 à 60).

Interpretation

Le TPIp avec la sulfadoxine-pyriméthamine semble avoir de puissants effets non paludéens sur le poids à la naissance. Des recherches supplémentaires sont nécessaires pour évaluer la dihydroartémisinine-pipéraquine administré mensuellement avec la sulfadoxine-pyriméthamine (ou un autre composé avec des effets non

paludéens) pour obtenir une meilleure protection contre les causes paludéennes et non paludéennes de faible poids à la naissance.

Financement

"Eunice Kennedy Shriver National Institute of Child Health and Human Development", "Bill and Melinda Gates Foundation", et "Worldwide Antimalarial Resistance Network".
