# Supplementary material for: Overall, anti-malarial, and non-malarial effect of intermittent preventive treatment during pregnancy with sulfadoxine-pyrimethamine on birthweight: a mediation analysis
Source: Lancet Glob Health. 2020 Jun 17;8(7):e942–53. doi: 10.1016/S2214-109X(20)30119-4 (PMC7303957; doi:10.1016/S2214-109X(20)30119-4)
Supplement: Supplementary appendix 2 [file mmc2.pdf]

THE LANCET
Global Health

Supplementary appendix 2

This appendix formed part of the original submission and has been peer reviewed. We post it as supplied by the authors.

Supplement to: Roh ME, ter Kuile FO, Rerolle F, et al. Overall, anti-malarial, and non-malarial effect of intermittent preventive treatment during pregnancy with sulfadoxine-pyrimethamine on birthweight: a mediation analysis. *Lancet Glob Health* 2020; **8:** e942–53.

Supplementary Appendix 1. Assessment of the association between mother's malaria infection status (mediator) and birthweight (outcome) to define the choice in the mediator.

Women were categorized into four groups that defined her malaria infection status based on the indication of whether she had peripherally detected malaria and/or placental malaria. Here, we define peripheral malaria as the detection of *P. falciparum* parasites in the maternal peripheral blood by PCR at any point between study drug initation to delivery. Placental malaria was defined as the indication of past or active infections by placental histopathology and/or the presence of parasites in the placental blood detected by microscopy, PCR, and/or LAMP.

From our adjusted analysis, we found that placental malaria (and not peripheral malaria) was associated with lower birthweights. Our findings support prior studies which suggests that the mechanism by which *P. falciparum* malaria increases the risk of low birthweight is via the sequestration of the *P. falciparum* parasite in the placenta[1,2]. Thus, we chose to define our mediator as **placental malaria**.

Mother's malaria infection status	N=1605	Mean Birthweight (SD)	Adjusted mean difference in birthweight[1]	
			Beta (SE)	p-value
No peripheral or placental malaria	584 (36.4)	3213 (437)	Ref	--
Peripheral, but no placental malaria	369 (23.0)	3173 (435)	43.1 (33.7)	0.20
No peripheral, but placental malaria	208 (27.7)	3129 (490)	-23.2 (28.6)	0.54
Peripheral and placental malaria	444 (13.0)	3024 (461)	-57.4 (35.5)	0.11

Note: SD=standard deviation; SE=standard error
[1] All models adjusted for the same covariates used in final mediation outcome models (e.g. randomized treatment arm, age at enrolment, household wealth, mother's education, gravidity, gestational age at enrolment, parasitemia at enrolment) and included study site as a fixed effect.

Supplementary Appendix 2. Directed acyclic graph depicting the relationship between random assignment to IPTp regimen ($IPTp_Z$) and birth outcomes as mediated by placental malaria infection during pregnancy. Subscript Z denotes randomization and C represents a vector of baseline mediator-outcome confounders (e.g. gestational age at enrolment, maternal age, presence of maternal parasitemia at enrolment, education, household wealth, and gravidity). The red path indicates the causal effect of IPTp on birthweight that is mediated by the prevention of placental malaria infection (i.e. antimalarial effect) and the blue path indicates the causal effect of IPTp on birthweight that is not mediated through placental malaria infection (i.e. non-malarial effect). Note: IPTp=intermittent preventive treatment; DP=dihydroartemisinin-piperaquine; SP=sulfadoxine-pyrimethamine.

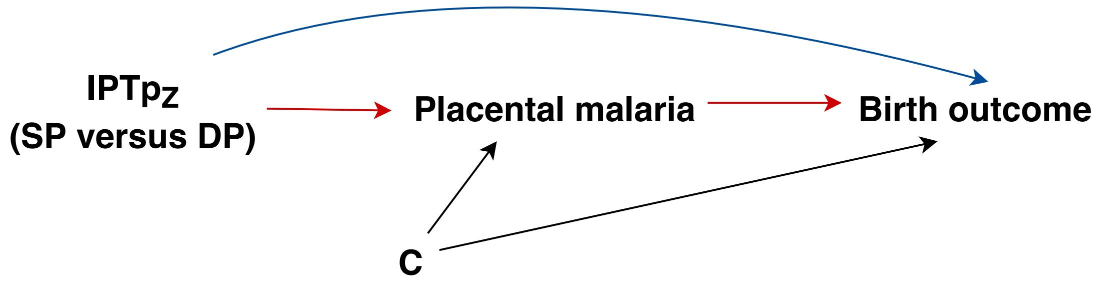

Supplementary Appendix 3. Details of causal mediation analysis methods.

For this study, we estimated the natural indirect (NIE) and direct (NDE) effects as described by Pearl et al.[1,2] The NIEs and NDE evaluate the mediated and non-mediated effects of the treatment on the outcome when the mediator takes on the value it would have naturally taken under specified counterfactual treatment values[1] (e.g. the NIE and NDE would estimate the relative mediated and non-mediated effect of IPTp with sulfadoxine-pyrimethamine versus IPTp with dihydroartemisinin-piperaquine on birth outcomes, had the risk of placental parasitemia in this study population been what it would have naturally been had women received either sulfadoxine-pyrimethamine or dihydroartemisinin-piperaquine).

To estimate total, direct, and indirect effects, we used the `mediation` package[3] in R. The associated `mediate()` function uses a potential outcomes framework[4] to estimate counterfactual values of the outcome under different treatment and mediator scenarios. This requires the specification of two models.

The first model represents the mediator as a function of the treatment and specified predictors (which in our case, were the covariates identified as mediator-outcome confounders). Predictions from this mediator model were used to estimate counterfactual mediator values for each woman: $M_{t=1}$ and $M_{t=0}$, where M_t=the counterfactual mediator value had the individual received treatment value t).

The second model fits the outcome as a function of the treatment, mediator, and mediator-outcome confounders. This fitted model was then used to predict four (possibly) counterfactual outcomes for each woman: $Y_{t=1,M(t=1)}$, $Y_{t=0,M(t=1)}$, $Y_{t=1,M(t=0)}$, and $Y_{t=0,M(t=0)}$; where $Y_{t,M(t)}$ is the outcome for specified counterfactual values of treatment (t=0,1) and mediator ($M_{t=0,1}$). Counterfactual mediator values predicted from the first model ($M_{t=1}$ and $M_{t=0}$) were used to predict counterfactual outcomes in the second model. These counterfactual values are then used to estimate the NDE = $\bar{Y}_{t=1,M(t=0)}$ - $\bar{Y}_{t=0,M(t=0)}$ and NIE= $\bar{Y}_{t=1,M(t=1)}$ - $\bar{Y}_{t=1,M(t=0)}$, where $\bar{Y}$ represents the outcome mean.

Mediation effects and 95% confidence intervals were estimated with a quasi-Bayesian Monte Carlo approach using 1,000 simulations. NIE and NDE risk ratios were calculated by modifying the functions to calculate NDE = $\bar{Y}_{t=1,M(t=0)}$ / $\bar{Y}_{t=0,M(t=0)}$ and NIE = $\bar{Y}_{t=1,M(t=1)}$ / $\bar{Y}_{t=1,M(t=0)}$. Effect modification of NIE and NDE were tested using the `test.modmed()` function, with p-values reported as $p_{difference}$.

Supplementary Appendix 4. Sensitivity analysis of the crude effect of IPTp regimens on active placental malaria. Active placental infections were defined as the presence of malaria parasites by placental tissue histopathology and/or the presence of parasites by microscopy or molecular methods in the placental blood.

	SP n/N (%)	DP n/N (%)	Active PM RR [95% CI]	p-value	Weight
All gravidae					
Kenya-STOPMiP	54/402 (13·4)	22/404 (5·5)	0·41 [0·25, 0·65]	<0·0001	40·0%
Uganda-BC1	17/94 (18·1)	2/84 (2·8)	0·13 [0·03, 0·55]	0·006	24·5%
Uganda-BC3	68/308 (22·1)	7/324 (2·2)	0·10 [0·05, 0·21]	<0·0001	35·6%
**Random effects model**			**0·19 [0·06, 0·54]**		**100·0%**
Heterogeneity: I^2 = 81%, p < 0·01					
Primigravidae					
Kenya-STOPMiP	27/144 (18·8)	11/125 (8·8)	0·47 [0·24, 0·91]	0·024	51·3%
Uganda-BC1	12/34 (35·3)	2/28 (7·1)	0·20 [0·05, 0·83]	0·027	15·6%
Uganda-BC3	27/80 (33·8)	5/71 (7·0)	0·21 [0·08, 0·51]	0·0001	33·1%
**Random effects model**			**0·31 [0·17, 0·57]**		**100·0%**
Heterogeneity: I^2 = 22%, p = 0·28					
Multigravidae*					
Kenya-STOPMiP	27/258 (10·5)	11/279 (3·9)	0·38 [0·19, 0·74]	0·005	--
Uganda-BC1*	5/60 (8·3)	0/56 (0·0)	--	--	--
Uganda-BC3	41/228 (18·0)	2/253 (0·8)	0·04 [0·01, 0·18]	<0·0001	--

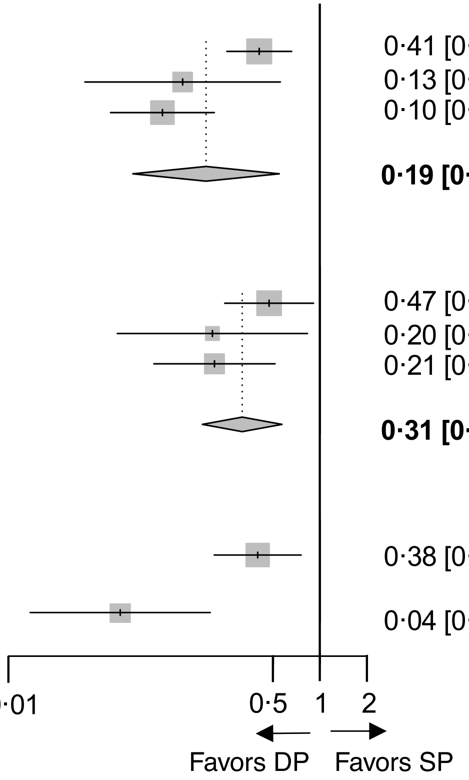

Note: ANC=antenatal care; CI=confidence interval; DP=dihydroartemisinin-piperaquine; PM=active placental malaria; RR=relative risk ratio; and SP=sulfadoxine-pyrimethamine.
* Relative risk ratio not calculated for Uganda-BC1 multigravidae and multigravidae random effects model as zero events of active placental infection occurred among Uganda-BC1 multigravid women randomised to dihydroartemisinin-piperaquine.

Supplementary Appendix 5. Sensitivity analysis of antimalarial (A) and non-malarial (B) effect estimates where the mediator is defined as active placental infections only. Active placental infections were defined as the presence of malaria parasites by placental tissue histopathology and/or the presence of parasites by microscopy or molecular methods in the placental blood. Panel A presents mediated/antimalarial effects and panel B presents non-mediated/non-malarial effects.

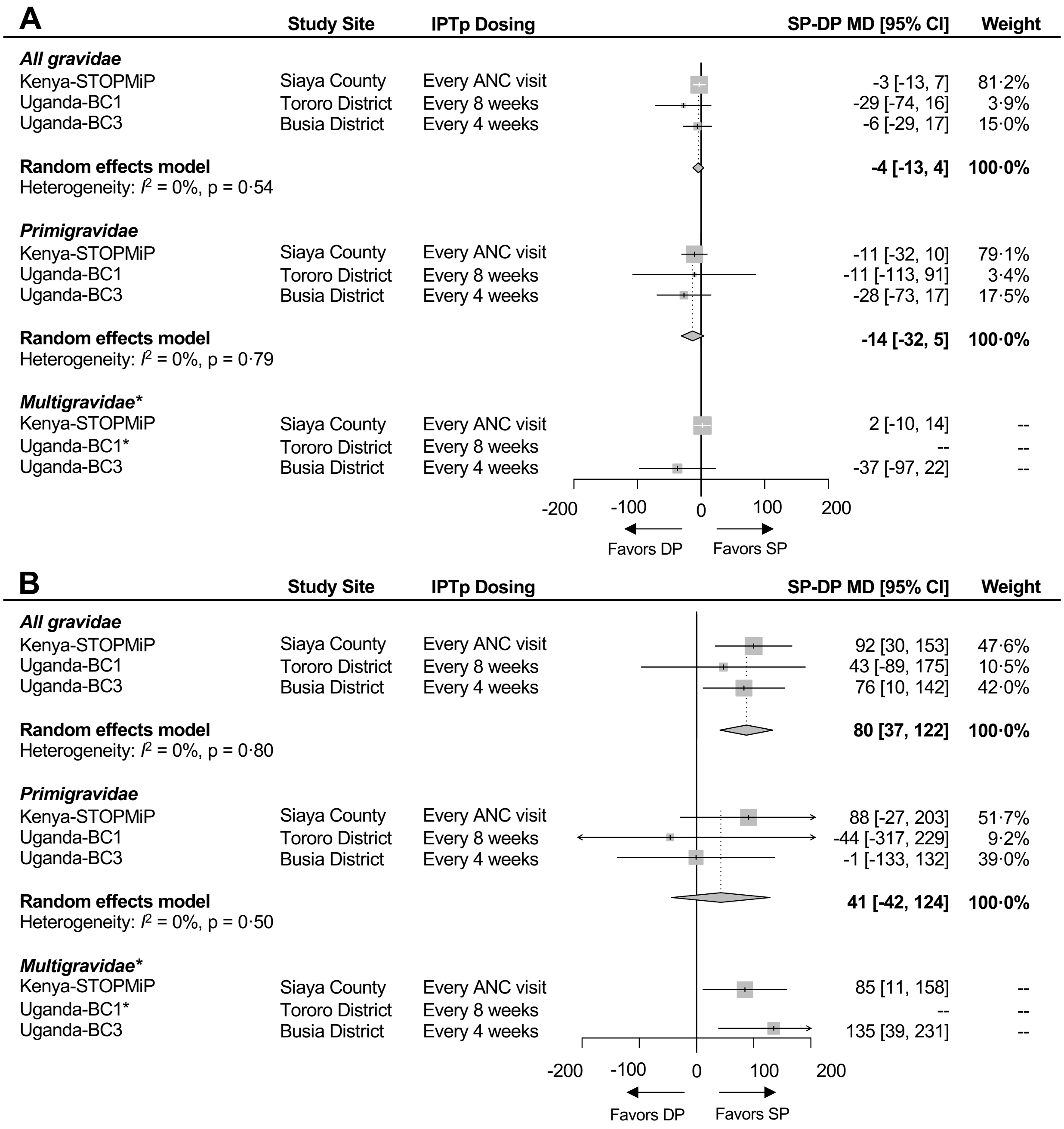

Note: ANC=antenatal care; CI=confidence interval; DP=dihydroartemisinin-piperaquine; IPTp=intermittent preventive treatment; MD=mean difference; and SP=sulfadoxine-pyrimethamine.
* Mediation effect estimates were not calculated for Uganda-BC1 multigravidae or for multigravidae random effects model as zero events of the mediator occurred among Uganda-BC1 multigravid women randomised to dihydroartemisinin-piperaquine.

Supplementary Appendix 6. Sensitivity Analysis to Assess the Degree of Unmeasured Mediator-Outcome Confounding Needed to Explain Away Indirect (Mediated)/Direct (Non-mediated) Effect Estimates.

Identification of direct and indirect estimates relies heavily on the following assumptions of mediator-outcome confounding:

(A1) There must not be any unmeasured confounding between treatment and outcome.
(A2) There must not be any unmeasured confounding between treatment and mediator.
(A3) There must not be any unmeasured confounding between mediator and outcome.

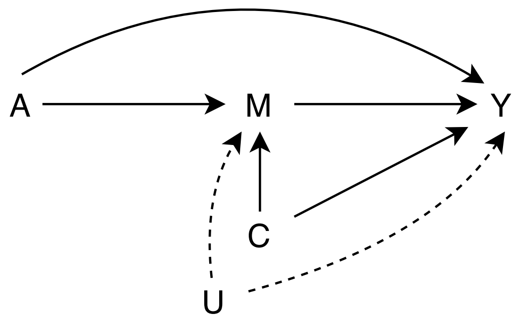

The relationship of these variables is depicted in the directed acyclic graph (DAG) above, whereby A=treatment, M=mediator, Y=outcome, C=measured confounders, and U=unmeasured confounders. We expect conditions A1-A2 to hold as a result of treatment randomization, but condition A3 may not. Here, we present sensitivity analyses to assess the robustness of our estimated direct and indirect effects on birthweight, in the presence of unmeasured mediator-outcome confounding.

Birthweight. Sensitivity analyses for the direct and indirect effects on birthweight were conducted using methods previously described by VanderWeele et al[5]. In brief, let B_{add} be a bias factor that denotes the difference between the observed effect estimate and the effect estimate we would have obtained had adjustment for the unmeasured confounder (U) been made. To estimate this bias factor (B_{add}), we used the formula, $B_{add}^{NDE} = \gamma_m \delta_m$ for the natural direct effect and $B_{add}^{NIE} = -B_{add}^{NDE} = -\gamma_m \delta_m$ for the natural indirect estimate, which estimates the effect of U on our outcome (Y), conditional on our treatment, mediator and measured outcome-confounders (γ_m) and the difference in the prevalence of U between treatment groups, conditional on mediator and measured mediator-confounders (δ_m). Sensitivity analyses assumed that the unmeasured confounder (U) was binary and that there was no interaction between treatment (A) and the unmeasured confounder (U).

Using the formulas above, we varied the sensitivity parameters (γ_m and δ_m) to quantify the extent of bias that an unmeasured confounder would be required to have to explain away completely the direct or indirect effect and to shift completely the confidence interval to include the null.

<u>Sensitivity parameters on the direct effect on birthweight.</u> Using the sensitivity analysis approach described above, we found that relatively large parameters of either γ_m or δ_m would be needed to explain away the relative non-malarial effect of sulfadoxine-pyrimethamine compared to dihydroartemisinin-piperaquine (see Figure below). Small differences in the prevalence of the unmeasured confounder between sulfadoxine-pyrimethamine and dihydroartemisinin-piperaquine would require very strong, and likely implausible associations between the unmeasured confounder and birthweight (e.g. if the difference in the prevalence of the confounder between IPTp groups (δ_m) is 0.05, the effect of the confounder on birthweight (γ_m) would need to be 1740 grams to explain away the observed direct effect). Equally, large differences in the prevalence of the unmeasured confounder between IPTp groups would be needed to explain away the observed relative non-malarial benefit of SP vs DP (e.g. if δ_m=0.95, γ_m=92 grams). Thus, it seems unlikely that the magnitude of the non-malarial benefit of SP observed in our analyses is entirely due to unmeasured mediator-outcome confounding.

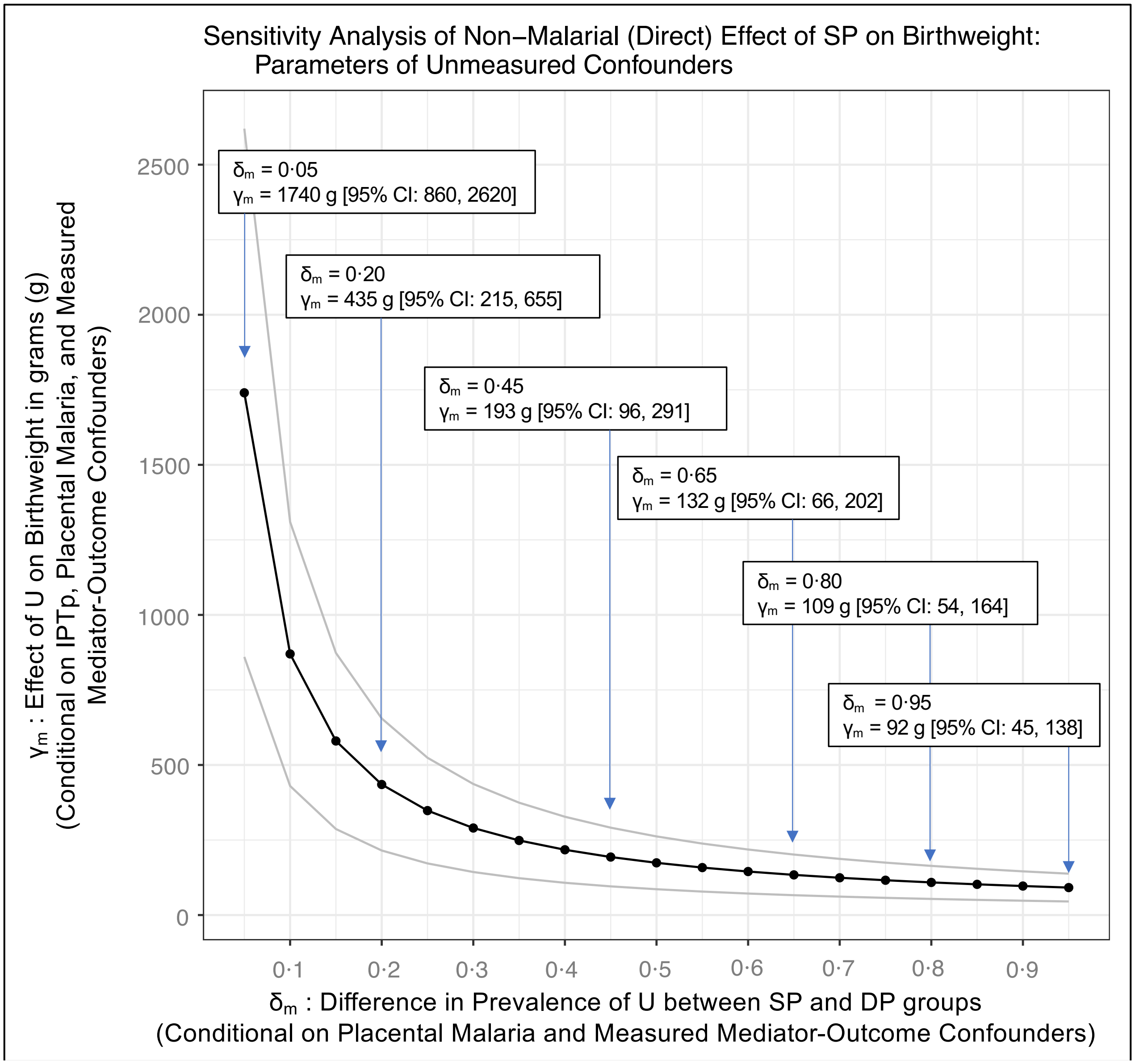

DP=dihydroartemisinin-piperaquine; SP=sulfadoxine-pyrimethamine; U=unmeasured confounder
Note: Point estimates are shown in black lines, and grey lines denote the upper and lower bounds of the 95% CI.

<u>Sensitivity parameters on the indirect effect on birthweight.</u> Sensitivity analyses were conducted on the observed antimalarial effects between IPTp regimens (see Figure below). We note that observed antimalarial effect, of which the confidence interval included the null, may be sensitive to unmeasured confounding, as moderate effect sizes of unmeasured confounder on birthweight or moderate to high differences in the prevalence of the unmeasured confounder between sulfadoxine-pyrimethamine and dihydroartemisinin-piperaquine groups could explain away the observed antimalarial effects on birthweight.

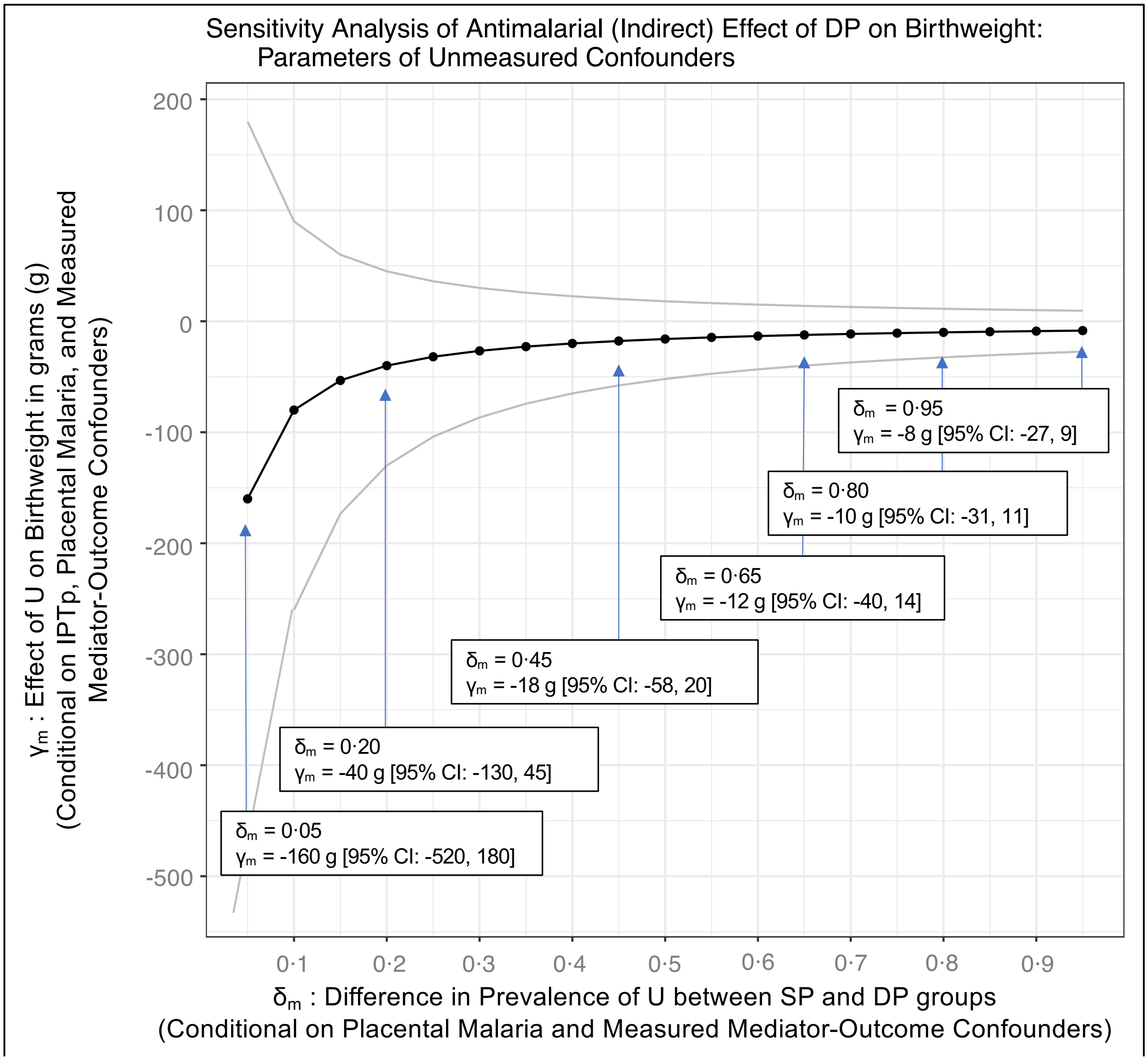

DP=dihydroartemisinin-piperaquine; SP=sulfadoxine-pyrimethamine; U=unmeasured confounder
Note: Point estimates are shown in black lines, and grey lines denote the upper and lower bounds of the 95% CI.

References

1. Pearl J. The causal mediation formula—a guide to the assessment of pathways and mechanisms. *Prev Sci* 2012; **13**(4): 426-36.
2. Robins JM, Greenland S. Identifiability and exchangeability for direct and indirect effects. *Epidemiology* 1992: 143-55.
3. Tingley D, Yamamoto T, Hirose K, Keele L, Imai K. Mediation: R package for causal mediation analysis. 2014.
4. Imai K, Keele L, Yamamoto T. Identification, inference and sensitivity analysis for causal mediation effects. *Stat Sci* 2010: 51-71.
5. VanderWeele T. Explanation in causal inference: methods for mediation and interaction: Oxford University Press; 2015.
